# Ventriculostomy-related infections in subarachnoid hemorrhage patients—a retrospective study of incidence, etiology, and antimicrobial therapy

**DOI:** 10.1007/s00701-016-3039-2

**Published:** 2016-12-07

**Authors:** Johan Widén, Britt-Marie Eriksson, Elisabeth Ronne-Engström, Per Enblad, Gabriel Westman

**Affiliations:** 1 Department of Medical Sciences, Uppsala University, 751 85 Uppsala, Sweden; 2 Department of Neuroscience, Uppsala University, Uppsala, Sweden

**Keywords:** Ventriculostomy, Meningitis, Subarachnoid hemorrhage, Antimicrobial therapy

## Abstract

**Background:**

This study was performed to investigate the incidence and etiology of ventriculostomy-related infections (VRIs) in patients with subarachnoid hemorrhage (SAH) and to assess adherence to local clinical guidelines regarding empirical antimicrobial therapy and diagnostic routines.

**Methods:**

A total of 191 consecutive SAH patients treated in the neuro-intensive care unit of Uppsala University Hospital between 2010 and 2013 were included retrospectively. Information regarding cerebrospinal fluid samples, bacterial cultures, ventriculostomy treatment, patient characteristics, and antibiotic treatment were collected from electronic patient records.

**Results:**

Eleven patients developed VRI, resulting in an incidence of 5.8% per patient, 5.4% per ventriculostomy catheter, and 4.1 per 1000 catheter days. Coagulase-negative staphylococci caused nine cases of VRI and *Klebsiella pneumoniae* and *Staphylococcus aureus* caused one each. Empirical VRI therapy was initiated on 97 occasions in 81 subjects (42.4%). Out of the 11 patients with VRI, four did not receive empirical antibiotic therapy before the positive culture result. The clinical actions performed after analysis of CSF samples were in line with the action suggested by the local guidelines in 307 out of 592 cases (51.9%).

**Conclusions:**

The incidence of VRI in our cohort was comparable to what has previously been reported. Coagulase-negative staphylococci was the most common agent. Our study demonstrates the difficulty in diagnosing VRI in SAH patients. Improved adherence to clinical guidelines could to some extent reduce the use of empirical antibiotic treatment, but better diagnostic methods and routines are needed.

## Introduction

Subarachnoid hemorrhage (SAH) is a severe condition, causing substantial morbidity and mortality [16, 35]. The management of patients with SAH is centered on the prevention of rebleeding by occluding the aneurysm and minimizing secondary brain insult by preventing and treating complications such as vasospasm, hydrocephalus, increased intracranial pressure (ICP), and infections. To monitor ICP and to treat secondary hydrocephalus, ventriculostomy catheters are often used. This management may increase the risk of infection, as microorganisms can colonize the device and spread to cerebrospinal fluid (CSF) and meninges to cause meningitis or ventriculitis [6]. These infections are called ventriculostomy-related infections (VRIs). Functional outcome and mortality after SAH is multifactorial and the exact impact of VRIs is not yet known. Several factors such as duration of ventriculostomy treatment [1, 2, 23, 26, 33, 37], irrigation of the ventriculostomy catheter [2, 7, 26], and presence of intraventricular blood [2, 23, 26, 37] have been shown to increase the risk of a VRI. The incidence of VRIs has varied significantly in previous studies, but a 2002 meta-analysis showed an incidence of 8.8% per patient treated with a ventriculostomy catheter [21]. Lumbar CSF drainage has been suggested as an alternative to ventriculostomy, possibly with a lower risk of intracerebral hemorrhage, vasospasm, and faster clearance of subarachnoid blood clots [18, 24, 36]. Lumbar drainage carries the disadvantage of not allowing reliable ICP monitoring, limiting its clinical usefulness. Whether increased use of lumbar drainage could affect VRI rates is not known.

The diagnosis of VRIs in the SAH setting is challenging as the underlying disease may cause fever, nuchal stiffness, headache, and lowered consciousness [27]. Moreover, interpretation of routine laboratory analyses of CSF such as cell count, glucose, albumin, and lactate is complicated by the presence of intraventricular blood and inflammation, either from the initial bleed or surgical interventions. This can affect the reliability of these parameters to indicate CNS infection [32]. Standard CSF bacterial cultures have long remained the gold standard for diagnosing VRIs but carry the disadvantage that they take several days to complete, making empirical treatment necessary in patients at risk. It has also been discussed whether standard cultures might have limitations in sensitivity, with reports of culture-negative but PCR-positive samples in patients with suspected VRI [3]. This has led to debate on how VRIs should be diagnosed and monitored and when empirical treatment should be considered. Several strategies have been proposed such as routine CSF cultures in all patients with ventriculostomy catheters, calculation of indices relating cell count in CSF to blood [30], and the analysis of CSF lactate as a reliable indicator of VRI [19, 25]. Hitherto, no consensus has been reached.

The aim of this study was to investigate the incidence and bacteriological etiology of VRI in patients with SAH that have been treated with a ventriculostomy catheter in the neuro-intensive care unit (neuro-ICU) of Uppsala University Hospital during the period 2010–2013. Further objectives were to investigate the extent of empirical antibiotic treatment for VRI and adherence to local guidelines for VRI diagnostics and treatment.

## Materials and methods

In total, 228 consecutive patients with spontaneous SAH treated with a ventriculostomy catheter were retrospectively screened for inclusion in the study. All patients were treated at the Department of Neurosurgery at Uppsala University Hospital between January 2010 and December 2013. The department has a catchment area of approximately 2 million inhabitants and is supported daily by infectious disease specialists. Patients were admitted to the neuro-ICU when a diagnosis of SAH had been radiologically verified, excluding patients considered terminally ill. In total, 37 patients fulfilled one or more exclusion criteria (Table 1), leaving 191 subjects for further analysis.

**Table 1:** List of patients excluded from the study

Reason for exclusion	Number of patients
Wrong diagnosis	4
Substantial neuro-ICU-care in another hospital	17
Management affected by other clinical study	16
Total	37

A ventriculostomy catheter was placed in all SAH patients not responding to commands. The catheter was placed through a frontal lobe burr hole and was fixated with sutures after at least 5 cm subcutaneous tunneling. No catheters with antimicrobial coating were used. Two grams of cloxacillin was administered intravenously at the start of surgery and further doses of 1 g were given every third hour during surgery. Clindamycin, 600 mg at the start of surgery and 300 mg every third hour, was used if subject was allergic to penicillin. A closed ventriculostomy system was used and CSF samples were collected upon clinical signs of infection such as onset of fever, unexplained rise in system inflammatory parameters, or deteriorating neurological performance. The catheters were not routinely exchanged.

Information was extracted from electronic patient records regarding age, gender, time of bleeding, aneurysm intervention, time of ventriculostomy placement, length of ventriculostomy treatment, volume of CSF drained, number of samples and cultures collected from CSF, antibiotic treatment, results of laboratory tests of CSF (cell counts, glucose, lactate, and albumin) and peripheral blood (white cell count, C-reactive protein (CRP), and plasma glucose) as well as the results of all bacterial cultures. Data from 758 occasions of CSF and blood sampling were collected. Antibiotic use directed against VRI was estimated by multiplying number of days of VRI treatment with standard VRI dosage of the drugs used. Antibiotic use was estimated in defined daily doses (DDD). Cultures were performed at the Department of Clinical Microbiology and Hospital hygiene and analysis of CSF was performed at the Department of Clinical Chemistry and Pharmacology, both at Uppsala University Hospital. Data regarding clinical parameters (Fisher grade [13], World Federation of Neurological Surgeons SAH-grade (WFNS) [28] and Glasgow coma scale motor response (GCS motor) [38]) on admittance were collected from electronic health records.

Clinical decisions regarding initiation of antibiotic treatment and CSF sampling for culture were registered and compared to the recommendations in local management guidelines (Table 2). VRI cases were defined as positive CSF culture along with inflammatory parameters above a pre-defined threshold in CSF. The definition of inflammation in CSF was the same as the criteria used in the local guidelines for when to send CSF for cultures (Table 2).

**Table 2:** Local guidelines for indications of CSF culture and empirical treatment

	Send CSF for bacterial culture*	Initiate treatment for VRI*
Corrected CSF polynuclear leucocytes**	>50 × 10^6^/l	>250 × 10^6^/l***
CSF lactate	>3.5 mmol/l	>4.0 mmol/l
CSF plasma glucose ratio	<0.4	<0.35
CSF albumin	>0.40 g/l	

*If one or several of criteria are fulfilled

**Calculated as CSF-polynuclear leucocytes - CSF-erythrocytes/1000

***Or a marked rise from previous sample (a definition of a marked rise is not given in the clinical guidelines. In our study, a marked rise was defined as a doubling of corrected polynuclear leucocyte count between two samples taken within 48 h, resulting in a value of >100 × 10^6^/l)

Statistical analysis was performed using R version 3.0.1 (The R Foundation for Statistical Computing. Vienna, Austria). Statistical comparisons were made using the non-parametric Mann–Whitney *U* test and Fisher’s exact test. The study was approved by the Regional Ethical Review Board in Uppsala.

## Results

### Epidemiology

Data regarding subject age, gender, clinical status on admittance according to GCS-motor and WFNS, radiological status according to Fisher grade, as well as data on length of ventriculostomy catheter treatment, volume of drained CSF, number of CSF samples, and aneurysm intervention are shown in Table 3. The VRI group had longer duration of ventriculostomy catheter treatment, larger CSF draining volumes, and more CSF samples drawn. There were no significant differences between the VRI and non-VRI groups regarding age, gender, aneurysm intervention, or clinical and radiological status on admittance.

**Table 3:** Patient characteristics. All data reported as medians

	No VRI	VRI	*p* value*
Age (years)	60	58	0.46
Gender (male:female)	56:124	3:8	0.77
GCS motor on admittance (1–6)	6	5	0.44
WFNS on admittance (1–5)	4	4	0.89
Fisher grade (1–4)	4	4	1.0
EVD (days)	12	17	0.00085
Drainage volume per day (ml)	71	153	0.0011
Number of CSF samples drawn	4	8	5.76e-05
Aneurysm intervention (clipping:coiling:none)	53:118:9	1:10:0	0.29

*Using Mann–Whitney *U* test. For gender and aneurysm intervention, Fisher’s exact test was used

### Incidence of VRI

In the 191 subjects included for analysis, a total of 205 ventriculostomy catheters were inserted. In 14 subjects, more than one ventriculostomy catheter was placed. The ventriculostomy catheters were kept for a median of 13 days, giving a total of 2683 catheter days. From the 758 CSF samples taken, bacteriological culture was performed on 453. In total, 26 cultures from 18 different patients presented with bacterial growth. There were seven patients (3.1%) with positive bacterial cultures that were classified as colonization of the catheter or contamination of the sample rather than VRI, due to lack of inflammatory signs in CSF. Out of 191 study subjects, 11 were considered to have developed VRI, resulting in an incidence of 5.8% per patient, 5.4% per ventriculostomy catheter, and an infection rate of 4.1 per 1000 catheter days.

### Microbiology

In the 11 cases defined as VRI, nine were caused by coagulase-negative staphylococci, one by *Klebsiella pneumoniae*, and one by *Staphylococcus aureus*. In the group of patients with positive CSF cultures that were regarded as contaminations, four were coagulase-negative staphylococci and there was one case of *Micrococcus* spp., alpha-hemolytic streptococci and diphteroids, respectively.

### Antimicrobial treatment

Empirical antibiotic treatment for bacterial meningitis was given to 81 (42.4%) of the patients on 97 occasions at some point during neuro-ICU care (Table 1). Eight of these patients (9.9%) had positive cultures and five represented VRIs according to the study definitions. Among the VRI cases confirmed by bacterial culture, four did not receive any empirical antibiotic treatment before the culture results and two subjects received therapy with only one drug (meropenem in one case and vancomycin in one case). In one case of VRI, the patient was admitted to another hospital shortly after the sample had been collected and no records of further treatment could be found. In 47 of 88 cases with a negative CSF culture, empirical treatment was discontinued within 72 h.

**Fig. 1:**
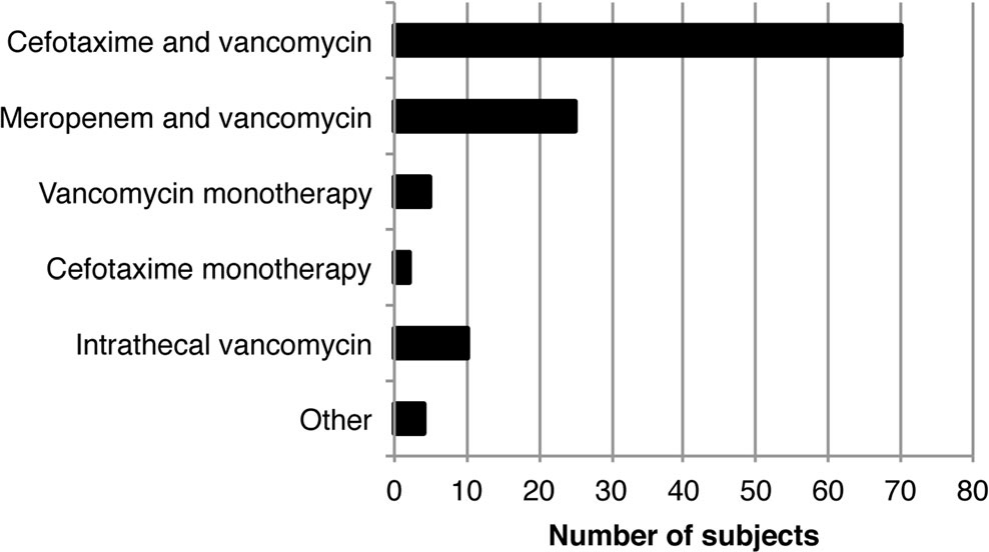
Antibiotic treatment. One subject may have received several types of treatment

The local first-line recommendation for empirical treatment of VRI is cefotaxime in combination with vancomycin. The second-line treatment is meropenem in combination with vancomycin. Cefotaxime, vancomycin, and meropenem were the most commonly used antibiotics in the study (Fig. 1). Due to suspected allergic reaction, second-line treatment with trimetoprim-sulfamethoxazole was given in one case. Penicillin was used in one case to treat alpha-hemolytic streptococci found in CSF culture. Moxifloxacin in combination with intrathecal gentamycin was used in one case to treat *Klebsiella pneumonia*e. In one case, due to poor CSF penetration of vancomycin, rifampicin was used.

In cases where sufficient data were available, the total amount of VRI therapy was calculated in defined daily doses (DDD). In 177 patients, the total antibiotic treatment directed against VRI was estimated as 1471 DDD. To evaluate the potential of a rapid diagnostic method for VRI for reducing unnecessary use of antibiotics in the neuro-ICU, the total amount of empirical VRI treatment, given in patients where a negative CSF culture resulted in discontinuation of therapy, was estimated. In these 34 cases, 398 DDD of empirical VRI therapy (27.1% of total VRI therapy) was given.

### Adherence to local guidelines

**Fig. 2:**
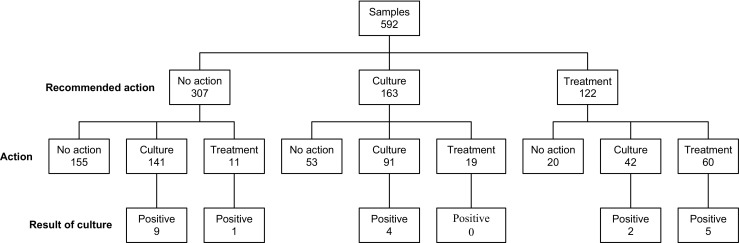
Results of CSF samples in relation to recommendations in clinical guidelines, actual clinical action, and result of cultures. Samples taken during VRI treatment excluded

To investigate adherence to local guidelines (Table 2), results from the CSF diagnostic work-up available at the time of decision were compared to the actual clinical decisions regarding CSF cultures and empirical VRI treatment (Fig. 2). For this analysis, all CSF samples collected during VRI treatment were excluded, resulting in a total of 592 CSF samples where the clinical action could be compared to the local guidelines.

The clinical action performed after each of the 592 CSF samples was in line with the action suggested by the clinical guidelines in 307 cases (51.9%). The deviations from guidelines included initiation of empirical VRI treatment, sending CSF for culture when the inflammatory signs were not sufficient and also omitting to do so when sufficient inflammatory signs were present. There were 307 samples that presented with such limited inflammatory signs that no action was recommended. In 141 (45.9%) of these, the clinician sent CSF for culture. This resulted in nine positive cultures, all regarded as contaminations according to the study definitions. In another 11 of the 307 cases with little to no inflammatory activity, VRI treatment was initiated. There were 163 cases where the recommendation stated that CSF should be sent for culture. In 53 (32.5%) of these cases, no action was performed. In 19 (11.7%) cases, VRI treatment was initiated. In the 122 cases when the inflammatory activity motivated VRI treatment, such treatment was initiated in 60 (49.1%) cases. There were 62 cases where no treatment was initiated even though sufficient inflammatory signs were present and in 20 of these no culture was performed.

## Discussion

In this study, 5.8% of the participants presented with a VRI, which is slightly lower than the 8.8% incidence found in a previous meta-analysis by Lozier et al. [21]. However, the incidence reported in the studies included in this meta-analysis used varying definitions of VRI that could affect the results. Most of the studies used the criteria established by Mayhall et al. [26], where VRI was defined as positive culture of CSF drawn from ventriculostomy catheter or by lumbar puncture. When using this definition in our study, the estimated VRI incidence is increased to 9.4%.

A number of studies have used more elaborate definitions of VRI requiring either a second positive culture with the same organism or inflammatory signs in CSF in conjunction with the positive culture for VRI diagnosis [2, 22, 23, 33]. However, due to the wide range of factors and cut-offs used to define the inflammatory response in CSF, no head-to-head comparison is possible. Surprisingly, out of all the studies included in the meta-analysis, the two with the highest reported incidence [2, 33] were in the group using stricter definitions and the combined incidence in the four studies using stricter definitions was 12.5%, higher than the average incidence of all included studies. This indicates that the variations in VRI incidence cannot solely be explained by differences in diagnostic criteria. Differences in epidemiology and ventricular catheter routines [9] may also have influenced the VRI incidences reported.

It is clear that a more robust and universal definition of VRI could enhance the quality of future research. Lozier et al. suggested definitions of contamination, colonization, possible VRI, VRI, and ventriculitis in their review and we believe our definitions are in line with those suggestions. However, using standard bacterial CSF culture as the gold standard for VRI definition could be problematic since the test does not strictly show the presence of bacteria, but rather their ability to multiply. This ability could be impaired if patients are treated with antibiotics penetrating to CSF, due to suspected VRI or other infections, which is common in all ICU settings. Until recently, no better alternative diagnostic test has been available but this might be subject to change in a near future.

The majority of VRIs in our study were caused by coagulase-negative staphylococci. Most studies present similar results [10, 27, 29, 31, 32, 37] but in some a broader range of pathogens were found, including varying rates of infections with Gram-negative rods, Gram-positive cocci, and fungi [1, 14, 23, 40]. Growth of coagulase-negative staphylococci in cultures is challenging for the clinician since they are often contaminants and are generally considered a low-virulence pathogen causing a relatively mild inflammatory response [4].

The extensive empirical VRI treatment compared to the relatively low incidence of VRI in our material illustrates the diagnostic difficulties and also gives and estimates the room for improvement in reducing antibiotic use. Furthermore, it is clear that clinical management guidelines are not always followed with respect to CSF cultures and initiation of empirical VRI treatment as additional factors are often taken into consideration. In the 353 cases when culture was performed on a subject not currently receiving VRI treatment, 141 (39.9%) were performed without the presence of inflammatory signs in the CSF. Using our definition of VRI, the cases with a positive culture result but without inflammation are classified as contaminations, but it is also possible that an unknown proportion of these represent actual VRIs with a limited or delayed inflammatory response. An explanation of the extensive use of CSF cultures could be that the clinical VRI guidelines do not include fever or changes in neurological status. However, these symptoms are highly unspecific and do not reliably indicate VRI [27]. It has also been shown that routine cultures from patients with ventriculostomy catheters do not identify infection earlier compared to an approach where sampling and cultures are performed on clinical indications [15, 32]. We believe a stricter adherence to guidelines regarding when to send CSF for culture could reduce over-diagnosing and unnecessary antibiotic treatment.

The large use of empirical VRI treatment is problematic from an ecological perspective since high antibiotic pressure can lead to selection of bacteria with pre-existing or inducible antibiotic resistance, affecting the risk and severity of future infections. Antibiotic treatment also increases the risk of complications such as *Clostridium difficile*-associated diarrhea (CDAD). Interestingly, even with this liberal use of empirical treatment, six of 11 cases did not receive full empirical VRI treatment before the positive result of the culture was obtained. Five of these cases were caused by coagulase-negative staphylococci and one case was caused by *Staphylococcus aureus*. In two of these cases, empirical treatment should have been initiated according to guidelines, and in four cases criteria for treatment were not met before the positive culture result. There were also 62 cases when no treatment was initiated even though criteria were met. In two of these cases, this delayed the initiation of VRI treatment. This implies that a stricter application of guidelines would result in an even higher incidence of empirical treatment, but could possibly reduce time to treatment for a limited number of patients with VRI. The guidelines state that the treatment should be discontinued if CSF cultures are negative after 48–72 h [8]. These routines have not been assessed in clinical trials but a retrospective analysis has shown that this approach is safe [40]. In our dataset, empirical treatment was sometimes continued for several days or even weeks although culture results were negative. This could partly be explained by the fact that a substantial part of all CSF samples were collected during antimicrobial treatment that could negatively influence the sensitivity of CSF cultures. However, a stricter approach in discontinuing empirical VRI therapy could help to reduce the total antibiotic load.

In our study, VRI was associated with longer duration of ventriculostomy treatment, larger volumes of CSF drainage, and a higher number of CSF samples drawn. The association to duration of ventriculostomy therapy has been previously demonstrated [1, 26, 33] and further shows the importance of rapidly removing ventriculostomy catheters when they are no longer needed. Longer ventriculostomy treatment is associated with larger drainage volumes and more sampling occasions, explaining these overlapping correlations to VRI. The association with increased sampling could also be due to the fact that additional samples are collected after a VRI diagnosis has been made, to monitor the effect of treatment. VRI can also elevate ICP and hence increase the need for CSF drainage.

The limitations in using routine CSF inflammatory parameters to diagnose and predict VRI, and the frequent deviations from clinical guidelines, indicate the need for better diagnostic methods. The 48–72 h required for a reliable negative result from CSF cultures is too long not to initiate empiric treatment if there is clinical suspicion of a VRI. Our estimations on antibiotic use for treating suspected or confirmed VRIs show that approximately one-third of the antibiotic use could be eliminated if faster diagnostic methods were available. PCR of conserved bacterial nucleic acid sequences such as 16S-rRNA is a rapid method that has become more widespread and available to the clinician. There have been reports of culture-negative cases of meningitis where PCR has been positive, indicating that cases of meningitis considered aseptic could have a bacterial etiology [3, 11, 20]. PCR, however, does not eliminate the problem of contamination, and frequent detection of environmental bacteria has been pointed out as a limitation [34]. Also, 16S-rRNA/rDNA do not specify the pathogen sensitivity or resistance to antibiotics, which limit the potential of reducing the use of broad-spectrum antibiotics. There are promising emerging technologies such as PCR ESI-MS, combining PCR with mass spectrometry for rapid detection of a broad range of pathogens [39] and the potential for this method has been previously assessed in a neurosurgical setting [5, 12]. It has also been reported that the method is reliable for identifying different species of staphylococci [17], which is interesting given the strong dominance of coagulase-negative staphylococci in VRI. To clarify the potential role of new diagnostic methods in the differentiation of aseptic and septic meningitis in patients with ventriculostomy, prospective clinical studies, where new methods are compared head-to-head with the current gold standard, are needed.
